# Changing the paradigm in health and care services: modern value chains using open innovation for the creation of new digital health solutions

**DOI:** 10.3389/fdgth.2023.1216357

**Published:** 2023-06-26

**Authors:** Joana Carrilho, Diogo Videira, Cláudia Campos, Luis Midão, Elísio Costa

**Affiliations:** ^1^ Competence Centre on Healthy and Active Ageing of the University of Porto, Porto, Portugal; ^2^ Faculty of Pharmacy of the University of Porto, Porto, Portugal; ^3^ Associate Laboratory i4HB- Institute for Health and Bioeconomy, UCIBIO-Applied Biomolecular Sciences Unit, Faculty of Pharmacy, University of Porto, Porto, Portugal

**Keywords:** open innovation, digital health, innovation, ecosystem, digital transformation

## Abstract

Digital Health is a subject of extensive discourse when considering its current and future significance. This significance arises from a convergence of various factors, including the escalating capabilities and cost-effectiveness of computing and communication technology, coupled with the mounting demands and challenges faced by healthcare systems. The integration of health and technology, when studied collectively with the purpose of addressing tangible real-world issues, holds the potential to generate substantial outcomes that greatly influence the provision of clinical and social care, thereby enhancing the overall well-being of both individuals and populations. In this sense, in this paper we propose a collaborative approach, using Open Innovation, where the most relevant stakeholders—health and care professionals, citizens and companies—work together to develop and validate innovative digital solutions for health and care. We have called this approach of value co-creation the **Collaborative Ecosystem**, and we focus specifically on the potential development of the Regional Ecosystem for Collaborative Innovation in Digital Health and Care, and the envisioned implications of its implementation in economic and social dimensions.

## Introduction

Demographic change is an acknowledged and extensively documented societal challenge with global recognition. The aging population and the escalating prevalence of chronic diseases have contributed to an increased need for healthcare, social services, and informal care. As a result, it is projected that healthcare and social care spending will rise by an average of 1%–2% of GDP until 2060. This surge in demand occurs amidst limited public resources allocated for health and social care, thereby presenting additional constraints (1–3).

Undoubtedly, there exists a distinct necessity to establish more sustainable frameworks for delivering health and social care. This imperative aligns with the ongoing process of Digital Health innovation, aiming to enhance the effectiveness and resilience of health and social care systems. Disruptive technologies associated with the concept of Digital Health, that provide digital and objective data accessible to both caregivers and patients, are triggering a cultural transformation that will lead to an equal level clinician-patient relationship with shared decision-making and the democratization of care (4).

The prevailing paradigm dictates that an increasing number of individuals experience poor health as a result of rapid lifestyle changes in contemporary society. The modern lifestyle provides easier access to energy-rich food while reducing opportunities for physical exertion. Maintaining good health now necessitates conscious efforts that extend beyond occupational activities or obtaining sustenance. In order to preserve good health, deliberate choices must be made regarding diet, work patterns, leisure activities, and rest.

Lifestyle-related illnesses exert a substantial burden on healthcare budgets in western economies, accounting for approximately 70% of expenditure. Moreover, the absolute value of these budgets is projected to escalate rapidly due to the aging population in many countries (5). Considering that healthcare budgets are already strained to their limits, it is evident that an alternative approach is urgently required. The advent of Digital Health holds the promise of facilitating improved self-care practices and averting or delaying the onset of severe diseases, offering a potential solution to these challenges.

Moreover, it is undeniable that we currently face new, urgent challenges posed by the global COVID-19 pandemic. We feel that now, more than ever, we have to provide citizens with solutions that allow them to maintain their health, even when access to medical care (in person) is unavailable. We wish to do so by “bringing care home”, meaning helping people access information about health-related topics, giving them tools to stay healthy, even with so many necessary lifestyle changes, helping them manage their pre-existing medical conditions (especially relevant for those suffering from chronic diseases that need constant monitoring), and allowing them to be evaluated and, if necessary, treated for new diseases.

In this paper we propose a collaborative approach, using Open Innovation, where the most relevant stakeholders—health and care professionals, citizens and companies—work together to develop and validate innovative digital solutions for health and care.

## Digital health overview

The definition of Digital Health is rather complex and authors include or exclude different aspects. But, overall, the term “Digital Health” encompasses a comprehensive range of elements, including information, processes, technologies, individuals, and systems. Together, these components empower individuals to make well-informed decisions regarding their health, take proactive measures to enhance it, and track their progress to identify the most effective approaches for their unique circumstances.

Based on emerging technologies for data gathering and sharing, including the ground-breaking advances in the Internet of things, artificial intelligence and machine learning, augmented and virtual reality, big data analytics, blockchain, and enhanced interfaces and displays, healthcare is being transformed. Every day, digital innovation emerges in various forms, including direct-to-consumer wellness products, remote patient monitoring, mobile applications (apps) and wearable technology, and innovative service models like telemedicine/telehealth, connected care, and virtual visits. Individuals can now share their biological and behavioural data with carefully chosen people and organizations within the healthcare system, allowing them to anticipate and address their health and care requirements.

Strategic design and cost-effective implementation of Digital Health innovations have the potential to enhance the “effectiveness, accessibility, and resilience of health and social care systems”. By doing so, they enable the emergence of novel service delivery business models (6).

According to the Committee on Quality of Health Care in America, the healthcare industry has consistently underinvested in appropriate use of information technology and it lags behind comparable knowledge-intensive industries by about 15 years (7). However, Digital Health presents a promising growth avenue for numerous companies. This is evidenced by the strong interest shown by global technology giants, including IBM and Google, who have allocated substantial investments in research and development specifically focused on health-related initiatives.

It is recognized that digital transformation of health and social care will be disruptive. Investment in the digital health sector is enormous, with a lot of new solutions coming to the market. For instance, after the explosion of the mobile health market in the COVID-19 pandemic, there were more than 350.000 health apps available to users with more than 250 health apps added daily to app stores (8).

This underscores the growing complexity and crowded environment that all healthcare stakeholders—including patients, providers, payers, industry, and regulators—are faced with. The primary challenge lies in discovering digital health solutions that deliver tangible value. Remarkably, there is currently no dependable mechanism available for identifying validated digital health solutions. Regulatory guidance and oversight are constrained, with enforcement primarily targeting companies that make exaggerated claims unsupported by evidence or those whose application failures pose risks to patient safety (9).

We are still facing the problem of enrolling patients and healthcare professionals in using digital solutions, which is one of the main barriers to the implementation of new digital services/products in real life. Moreover, to effectively develop, integrate, and implement digital health technologies, a significant departure from conventional, single-disciplinary approaches in academia and clinical settings is necessary. Embracing the potential of these technologies to revolutionize healthcare and enhance well-being calls for a fresh perspective on science and health research methods.

## Open innovation

Open innovation is defined as “the use of purposive inflows and outflows of knowledge to accelerate internal innovation, and expand the markets for external use of innovation, respectively. [This paradigm] assumes that firms can and should use external ideas as well as internal ideas, and internal and external paths to market, as they look to advance their technology” (10).

Building from the aforementioned concept of individual empowerment in the management of one's own health, we believe that there are two key concepts that define a common thread behind all actions on Open Innovation in the health and care sector: “Validation” and “User-centred”.

Validation is the process of gathering evidence and learnings around business ideas through experimentation and user testing, in order to make faster, informed, de-risked decisions.

Notwithstanding the irreplaceable role of fundamental research, we can no longer afford to create solutions for the sake of novelty generation. Nowadays, new solutions must place users at the core of the development process, if they wish to successfully enter the market and reach their intended goal. In that sense, we have in recent years witnessed the rise of Testbeds and Living Labs.

Living Labs are defined as “user-centred, open innovation ecosystems based on a systematic user co-creation approach integrating research and innovation processes in real life communities and settings” (11). In practical terms, Living Labs prioritize citizen engagement in the innovation process, thereby demonstrating their capacity to effectively adapt new concepts and solutions to the distinct requirements, aspirations, and creative potentials of local contexts and cultures.

There are several references to the importance of Living Labs in the EU's strategy policy documents (12–15), highlighting the relevance of human and social aspects for better design and implementation of Research, Development and Innovation (RDI) projects.

The underlying principle behind public intervention in Health, supported by the EU, combines the ambition to advance the technological frontier for the betterment of community well-being with the practical need to transform research, development, and innovation (RDI) outcomes into market-oriented products and services. These guiding principles not only influence the research agenda of Horizon Europe but also serve as a source of inspiration for RDI provisions within Cohesion Policy and Territorial Cooperation Programmes for the period 2021–2027. This is particularly evident in the adoption of Smart Specialisation Strategies by all EU regions and member states, which provide a framework for implementing the EU Digital Agenda at the local level. In this context, Living Labs can be somehow thought of as a transversal, technology-driven sample market, meeting the requests of the European Council and the European Parliament for “strengthening synergy between EU support policies in the area of research and innovation” and “placing regions and cities as leading actors in Europe's innovation strategies” (11).

It becomes clear from this excerpt that these kinds of initiatives are at the top of the priority list for European Union-supported projects.

## A new approach

Consequently, being aware of the existing demand for the increase of Digital Health solutions and the support that can be expected from the European Union guidelines for the upcoming years, we have conceptualized a new approach for the development of the health and care ecosystem of innovation and service provision.

As previously demonstrated, the increase in life expectancy and sedentary lifestyles have led to an alarming raise in the prevalence of chronic diseases and multi-morbidities. In order to lighten the burden on healthcare systems, we need to empower citizens to take upon themselves to manage their conditions autonomously.

This will also bring us closer to the ultimate goal of having truly personalized healthcare, as everyone will be able to knowingly select and employ whatever solutions fit their individual needs the best.

The best set of tools to achieve this is Digital Health. If we wish to ensure the success of this paradigm shift, we need to make sure that the technologies being developed are (a) fit to the real challenges of end-users and (b) user-friendly, to allow for significant adherence from the target market.

In this context, our vision is to improve health and wellbeing for everyone, by accelerating the development and adoption of appropriate Digital Health solutions. More specifically, we aim to be at the forefront of research in co-creation, development, validation and adherence promotion of disruptive innovative solutions, that provide digital and objective data accessible to both caregivers and patients, which can trigger a cultural shift in patient-clinician interactions, leading to more balanced relationships with shared decision-making and the democratization of care.

We need to find ways to co-create value for all relevant actors in society—whether it is economical value for companies and healthcare providers, social and scientific value for health and care professionals, or personal value—through personalized Health and Quality of Life improvement, and individual empowerment—for patients and citizens.

**Figure 1:**
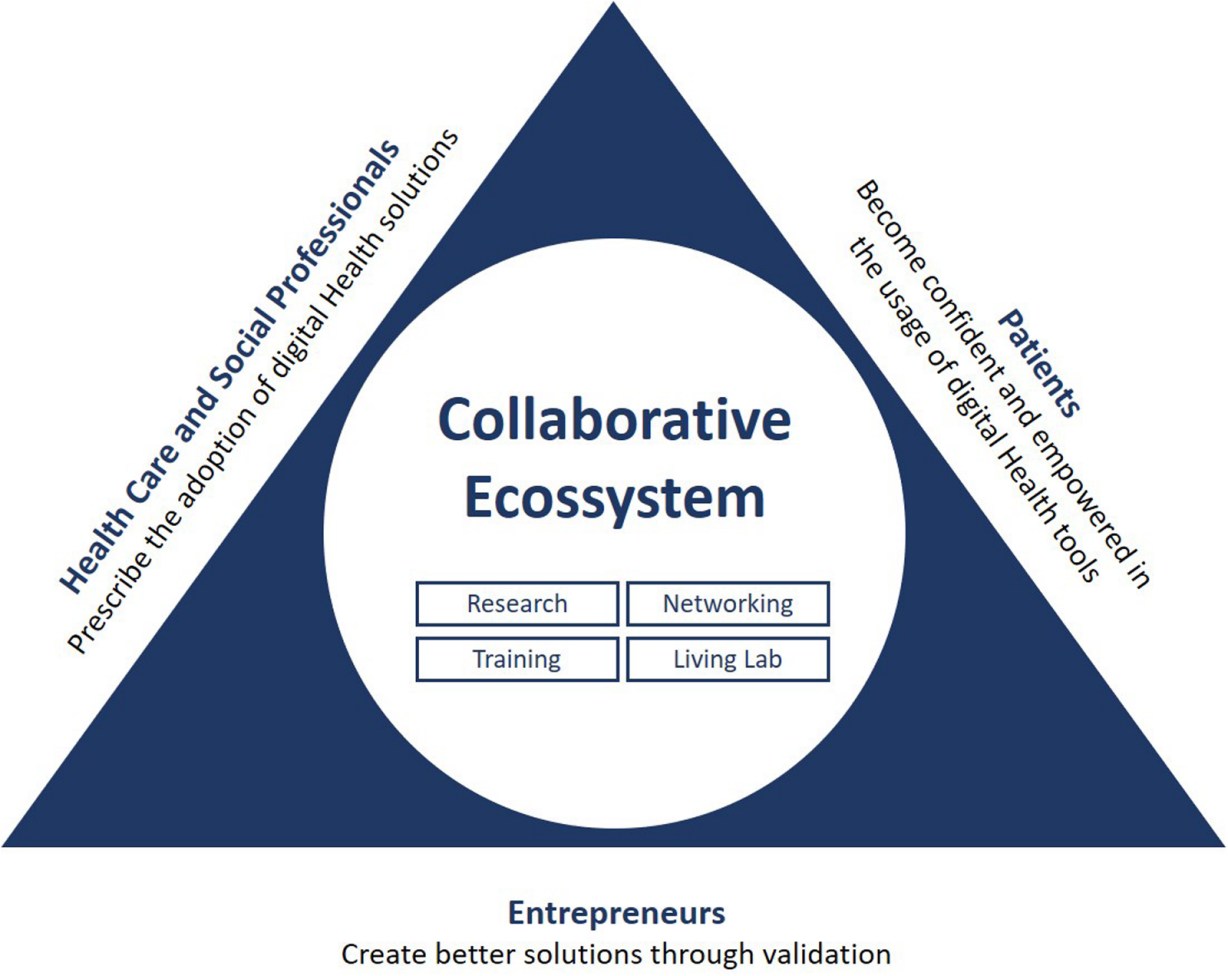
The collaborative ecosystem activities and their impact on relevant stakeholders.

Our positioning and strategy for the pursuit of this goal is to develop activities to contribute to digital transformation of health and care, acting at 3 different levels (entrepreneurs, health and social care professionals, and citizens/patients), as shown in Figure 1.

For each of these key stakeholder groups we have identified the main challenge and the expected long-term impact of our intervention. In detail, the challenges we need to tackle are:

- Citizens and Patients—lack of digital and health literacy, leading to a lack of trust in the solutions.
- Entrepreneurs—lack of awareness of the benefits of co-creation; insufficient knowledge or misconceptions regarding the right methodologies for end-user engagement.
- Health and Social Care Professionals—lack of digital literacy; lack of incentives for innovation.

Thus, we come to the core of our approach: the Collaborative Ecosystem.

Going beyond the Living Lab, we wish to not only engage the stakeholders in the innovation process chain, but also to generate knowledge that allows us to develop the best methodologies and protocols to do so, and mobilize formal and informal training initiatives that guarantee the success of the solutions and user long term adherence.

Allow us to deconstruct the concept of a Collaborative Ecosystem.

On the one hand, “Ecosystem” elicits the analogy to the biologic concept of the “system consisting of all the organisms and the physical environment with which they interact”. We bring this into fruition in our context by taking in consideration all the relevant players, whether individual or institutional, but also the surrounding conditions, features and uniqueness of our particular reality.

This also means that the composition of the Collaborative Ecosystem is dynamic and adaptative to the changes in the context, whether in demography, economy, regulatory framework, or others.

On the other hand, Collaborative, as the name says, implies that we are bringing together different segments of society and institutions, in a way in which their respective strengths and expertise complementarily come together to generate value for the whole.

Our proposition for a pilot implementation of this concept, shaped by the environment, needs and existing abilities of the region encompassing the North of Portugal and the neighbouring Galicia region of Spain, is called the Regional Ecosystem for Collaborative Innovation in Digital Health and Care.

We have designed this initiative to include academic partners (such as Universities and research centres), health and care providers, patient organizations, regional authorities and business creation organizations (such as incubators/science and technology parks).

The main pillars of activity of the Collaborative Ecosystem are:

1. Network development—build, segment and take advantage of the most comprehensive pool of end-users, on one side, and technology providers, on the other.
2. Research—shed new light on the factors surrounding technology acceptance and uptake by users; develop new interfaces and user-machine interactions to enhance usability; and create improved methodologies and guidelines for open innovation.
3. Living Lab activities—co-creation and validation of Digital Health solutions.
4. Training—in Digital skills for citizens and health and care professionals, in Health literacy for citizens, in Business Development for researchers.

By building an ecosystem attractive to European industry and policy-makers, the Collaborative Ecosystem has the potential to develop value-based business models to open and scale-up the market for digital solutions. It can also provide key recommendations for the far-reaching deployment of innovative digital health and care solutions, supporting and extending healthy and independent living at the European level.

The Collaborative Ecosystem partners recognise an opportunity to achieve, in collaboration, beyond state-of-the-art applied knowledge and digital solutions to improve the quality of life, wellbeing and care of users and carers, leading to a better and improved framework for sustaining healthy lives, and putting in place policies that would, otherwise, be unfeasible and too costly to pursue individually, particularly for business-oriented partners (care service providers and technology providers).

The Collaborative Ecosystem proposal is organised to facilitate and require strong interdisciplinary knowledge sharing among researchers, users, developers, and health and social practitioners. Combining technical and social sciences disciplines, this Collaborative Ecosystem is positioned to produce outputs based on active contributions of all partners.

Within the Euroregion of North of Portugal-Galicia, this could be the first time such an endeavour is taken at a major scale, with so many relevant players in the health and social sectors coming together to create added-value solutions.

## Discussion

To launch our endeavour, we will draw lessons from analogous initiatives throughout Europe. Namely, we have identified Trentino eHealth Ecosystem (16). Based upon this initiative's findings, we have realised that having a multitude of very different stakeholders in our ecosystem is not only not an hinderance, but actually an added-value to the success of the ecosystem. According to the authors of this study, the interaction between technology/service suppliers and patients generates value co-creation and innovation “if new resources, new uses of technology and new institutions are created”. By putting in place the conditions for this exchange to happen in a sustainable manner over time, we are guaranteeing that a new way of service delivery in health and care is being created, with sustainable value co-creation for all the parties involved.

According to the same authors, the main challenge in developing an initiative that actually generates value for its users, in a measurable way, is the lack of digital competences, that is an obstacle to the adoption of technology.

Another example of a network-approach (adjacent to the ecosystem we describe) is Service Design for Value Networks, and specifically its application to the design the Portuguese National Healthcare Record service (17). According to the authors of this study, normal approaches to value networks are based on a univocal relation between provider and users (even if these are different segments, whether or not connected—a *many-to-one* design), rather than parity approach of interdependent actors—*many-to-many*. This is a challenge when we plan to implement this type of model on a complex web of relationships such as the health and care sector.

Several other authors (18, 19), when referring to the main challenges to the successful implementation of a Digital Health Ecosystems reiterate that the main challenges to be faced are the lack of digital health literacy, self-management and collaboration in “the prevention, control, and alleviation of potential problems”. The latter is especially true when it comes to the need to keep stakeholders engaged in the long-term. Short-term sporadic joint actions are relatively common, but in order to maintain an ecosystem viable and functioning as such, we need that the multiple actors continue to interact with each other towards the development of common goals.

As such we, have integrated in our approach some tools to prevent these pitfalls, such as: (a) the implementation of training in Digital and Health Literacy, for both citizens and healthcare professionals; (b) the creation of a partnership where representatives of the different sectors of society and stakeholders in the creation of value in Digital Health have equal opportunity to shape the activities of the ecosystem; (c) the establishment if the Collaborative Ecosystem as an umbrella organization, that will be able to leverage its joint resources, the added value of the collaboration and the visibility of its dimension to raise funding that can then be used by other actors in the region, thus ensuring their engagement in the joint development efforts.

The next steps for the launch of the Collaborative Ecosystem are the creation of this new organization, where the main actors identified (academic partners, health and care providers, patient organizations, regional authorities and business creation organizations) will act as shareholders, thus ensuring transparency, accountability and commitment to the common goals, above individual institutional policies and priorities. The new umbrella organization will be the main representative of the interests of the different stakeholder groups and will be mandated to put in place the activities previously described.

There are several support and funding schemes, both at national and European level that can be leveraged for the launch of the initiative. However, we intend on being fully sustainable and self-funded. This choice is made following our experience in witnessing that initiatives supported by calls for public funding seldom continue to exist past the funding period, for lack of incentives for the implementing consortia to pursue long-term sustainability. That is why we plan to have the founding partners commit their own resources and develop profitable activities, in order to ensure the independency of the Collaborative Ecosystem from any contextual and policy paradigms.

It is expected that the Collaborative Ecosystem contributes in the short term to improve the (1) collaborative living-lab activities on Digital Health innovation, (2) number of validated digital solutions developed in co-creation with end-users, as a way to improve acceptance and adherence to technology; (3) end-user awareness to health and wellbeing issues, and digital technology use; (4) citizens' knowledge about the current available solutions for the challenges they face; (5) technology readiness level of the research produced by academia; (6) partnerships between academia and industry.

In the medium-term we expect to improve (1) the sense of empowerment and ownership of digital solutions by end-users; (2) the market's confidence in the available products/solutions, boosting the economical relevance of Digital Health solutions; (3) the competitiveness in the region, through the attraction of foreign business.

In the long-term Collaborative Ecosystems are the way to best improve the (1) overall quality of life of citizens, through the adoption of Digital Health innovative solutions; (2) public involvement in research by influencing research topics and directions, towards more applied and solution driven projects; (3) companies' and researchers' accountability, as transparency is present throughout the entire innovation chain.

We envision that, in the future, Digital Health innovation will be prioritized through dedicated bodies and mechanisms for governance. This will be translated into national Digital Health strategies or similar strategic frameworks. These strategies will be incorporated into national health strategies and actively employed to steer advancements and expedite progress towards the health-related objectives outlined in the Sustainable Development Goals. Furthermore, they will play a crucial role in facilitating the digital transformation of health systems.

The predicted outlasting legacy of the Collaborative Ecosystem will be the establishment, adoption and application of minimum standards for Digital Health solutions; the implementation of a mechanism facilitating personalized feedback for assessing the efficacy of Digital Health tools and services; and the development of comprehensive guidance lines on personalized medicine.

## Data Availability

The original contributions presented in the study are included in the article, further inquiries can be directed to the corresponding author.

## References

[1] European Commission. The 2018 ageing report: economic and budgetary projections for the EU member states (2016-2070) (2018). Available at: https://ec.europa.eu/info/publications/economy-finance/2018-ageing-report-economic-and-budgetary-projections-eu-member-states-2016-2070_en.

[2] United Nations. World population ageing 2019 highlights (ST/ESA/SER.A/430) (2019). Available at: https://www.un.org/en/development/desa/population/publications/pdf/ageing/WorldPopulationAgeing2019-Highlights.pdf.

[3] World Health Organization. World report on ageing and health (2015). Available at: https://www.who.int/ageing/events/world-report-2015-launch/en/.

[4] Meskó B Drobni Z Bényei É Gergely B Győrffy Z. Digital health is a cultural transformation of traditional healthcare. mHealth. (2017) 3:38. 10.21037/mhealth.2017.08.07 29184890 PMC5682364

[5] UK Government’s Foresight Programme. Tackling obesities: future choices (2007). Available at: https://assets.publishing.service.gov.uk/government/uploads/system/uploads/attachment_data/file/287937/07-1184x-tackling-obesities-future-choices-report.pdf.

[6] Black AD Car J Pagliari C Anandan C Cresswell K Bokun T The impact of eHealth on the quality and safety of health care: a systematic overview. PLoS Med. (2011) 18:8. 10.1371/journal.pmed.1000387 PMC3022523 21267058

[7] Institute of Medicine (US) Committee on Quality of Health Care in America. Crossing the quality chasm: A new health system for the 21st century. US: National Academies Press (2001). 25057539

[8] IQVIA Institute for Human Data Science. The growing value of digital health: evidence and impact on human health and the healthcare system (2021). Available at: https://www.iqvia.com/-/media/iqvia/pdfs/institute-reports/the-growing-value-of-digital-health.pdf.

[9] World Health Organization. Global diffusion of eHealth: making universal health coverage achievable. World Health Organization (2018). Available at: https://www.who.int/goe/publications/global_diffusion/en/.

[10] Chesbrough H. Open innovation: a new paradigm for understanding industrial innovation. In: Chesbrough WV Chesbrough WV, editors. Open innovation: Researching a new paradigm. Oxford, UK: Oxford University Press (2006). p. 1–12.

[11] European Network of Living Labs. The living labs methodology handbook (2007). Available at: https://www.living-labs.net/documentation/2007_living_labs_methodology_handbook.pdf.

[12] Helsinki Manifesto, Finland’s EU Presidency (2006). Available at: https://www.scribd.com/document/290101063/Helsinki-Manifesto-201106.

[13] European Commission’s Joint Research Center. Living labs for regional innovation ecosystems (n.d.). Available at: https://s3platform.jrc.ec.europa.eu/documents/20182/138085/Living+labs+for+regional+innovation+eco%20systems_update.pdf/7197a890-a0c2-4db6-9e7a-58fd7f63e20d.

[14] European Parliament. Digital agenda for Europe (2020). Available at: https://www.europarl.europa.eu/factsheets/en/sheet/64/digital-agenda-for-europe.

[15] European Commission, Joint Research Centre. Smart specialisation platform (n.d.). Available at: https://s3platform.jrc.ec.europa.eu/what-is-smart-specialisation-.

[16] Botti A Monda A. Sustainable value co-creation and digital health: the case of trentino eHealth ecosystem. Sustainability. (2020) 12(13):5263. 10.3390/su12135263

[17] Patrício L de Pinho NF Teixeira JG Fisk RP. Service design for value networks: enabling value cocreation interactions in healthcare. Serv Sci. (2018) 10:76–97. 10.1287/serv.2017.0201

[18] Benis A Tamburis O Chronaki C Moen A. One digital health: a unified framework for future health ecosystems. J Med Internet Res. (2021) 23(2):e22189. 10.2196/22189 33492240 PMC7886486

[19] Mackey T Fornazin M Fornazin M. Exploring the use of digital health innovations to improve health equity: an international perspective. Popul Med. (2023) 5(Supplement):A654. 10.18332/popmed/164003

